# Wheat Germ Agglutinin as a Potential Therapeutic Agent for Leukemia

**DOI:** 10.3389/fonc.2019.00100

**Published:** 2019-02-21

**Authors:** Bradley Ryva, Keman Zhang, Abhishek Asthana, Derek Wong, Yorleny Vicioso, Reshmi Parameswaran

**Affiliations:** ^1^Department of Pathology, School of Medicine, Case Western Reserve University, Cleveland, OH, United States; ^2^Division of Hematology/Oncology, Department of Medicine, School of Medicine, Case Western Reserve University, Cleveland, OH, United States; ^3^The Case Comprehensive Cancer Center, Case Western Reserve University School of Medicine, Cleveland, OH, United States

**Keywords:** WGA, leukemia, therapy, lectin, GlcNAc

## Abstract

Dietary lectins are carbohydrate-binding proteins found in food sources. We used a panel of seven dietary lectins to analyze cytotoxicity against hematological cancers. Wheat germ agglutinin (WGA), even at low doses, demonstrated maximum toxicity toward acute myeloid leukemia (AML) cells. Using AML cell lines, we show time- and dose-dependent killing by WGA. We also show that low doses of WGA kills primary patient AML cells, irrespective of subtype, with no significant toxicity to normal cells. WGA caused AML cell agglutination, but failed to agglutinate RBC's at this dose. WGA, primarily, binds to *N*-acetyl-D-glucosamine (GlcNAc) and is also reported to interact with sialic-acid-containing glycoconjugates and oligosaccharides. After neuraminidase pre-treatment, which catalyzes the hydrolysis of terminal sialic acid residues, AML cells were less sensitive to WGA-induced cell death. AML cells were also not sensitive to succinyl-WGA, which does not react with sialic acid. Incubation with LEL lectin, which recognizes GlcNAc or SNA, which binds preferentially to sialic acid attached to terminal galactose in α-2,6 and to a lesser degree α-2,3 linkage, did not alter AML cell viability. These data indicate that WGA-induced AML cell death is dependent on both GlcNAc binding and interaction with sialic acids. We did not observe any *in vitro or in vivo* toxicity of WGA toward normal cells at the concentrations tested. Finally, low doses of WGA injection demonstrated significant *in vivo* toxicity toward AML cells, using xenograft mouse model. Thus, WGA is a potential candidate for leukemia therapy.

## Introduction

Lectins, carbohydrate-binding proteins, have been well characterized for more than 40 years (1). Because they are present in many of our major staple foods, such as wheat, potato, soy, and tomato, they play an important role for humans (2). Their true biological function is as a pesticide and anti-fungal, preventing disease from spreading and killing the plant or organism (3). Lectins, also present in animals and fungi, are classified by evolutionary origin, three-dimensional structure, and binding specificity (4, 5). In the last 10 years, with technological improvements in protein structural analysis, lectins have been organized into seven families. Most lectins are within the legume, chitin-binding (hevein domain), type 2 ribosome-inactivating, and monocot mannose-binding lectin families; but there are also jacalin-related, amaranthin, and Cucurbitacea phloem families (6).

Lectins have wide-ranging biological activity at cellular, tissue, and organism levels. *In vitro*, it has been demonstrated that incubation with lectin from red kidney bean lead to T cell proliferation and increased cytokine production (7). Haas et al. demonstrated that certain dietary lectins can cause IL-4 and IL-13 release from basophils (8), while Gong et al. demonstrated that plant lectins can activate NLRP3 inflammasome in macrophages, although at concentrations outside of normal physiological conditions (9). Recently it has been shown that certain lectins can activate toll-like receptors (TLRs) in a distinct, yet comparable, fashion to activation by pathogens. Specifically, the lectin ArtinM leads to TLR activation, resulting in increased pro-inflammatory cytokine release (10). Since their first isolations, lectins have been known to agglutinate cells, including red blood cells (11). When lectins are ingested, they have effects on tissues and organs, partially because they are not digested by gut enzymes (12) but pass through the gut wall and enter the circulation (13, 14). Lectins have also been shown to cause gut inflammation and have been potentially linked to autoimmune disease, such as rheumatoid arthritis (15). Besides this biological activity toward normal tissues, lectins have been shown to exhibit specific effects on cancer cells.

Many lectins have demonstrated cytotoxic and anti-proliferative effect on cancer cells. In the early 1980s, it was shown that lectin from *Griffonia simplicifolia* administered to mice *in vivo* was cytotoxic toward ascites tumor cells (16). Miyoshi et al. showed that rice bran agglutinin (RBA) caused apoptosis and cell cycle disruption on human U937 monoblastic leukemia cells (17). Lectins like *Concanavalin* A, *Griffonia simplicifolia* (GSA-1A4), and *Phaseolus vulgaris* were shown to be toxic toward melanoma cell lines (18). Finally, Wang et al. looked at various lectins and their effects on cancers of the liver, chorion, skin, and bone. They determined that lectins from mushroom, soybean, and potato had varying impacts on these cell lines (19). Of the lectins tested, wheat germ agglutinin (WGA) had the most profound cytotoxic effects against these cell lines.

WGA, the lectin derived from wheat germ, binds specifically to *N*-Acetyl-D-glucosamine (GlcNAc). It has been reported that WGA also binds *N*-acetyl-neuraminic (sialic) acid; however more recently it has been characterized as interacting with sialic acid residues on glyconjugates and oligosaccharides (20). WGA is one of the most characterized and studied lectins. While studying the effect of WGA on normal gut epithelium, it was determined that WGA can bind the apical side of gut-like cells and alter the cell membrane permeability (21). Pellegrina et al. also quantified whether the amount of wheat consumed in the normal diet is toxic. They concluded that in order to reach toxic levels more than 1 kilogram of uncooked pasta would need to be consumed in one meal (21). Despite the limited toxicity to normal tissues, it has been shown that WGA is toxic to pancreatic, liver, bone (osteosarcoma), and skin (melanoma) cancer in low doses (18, 19, 22). WGA causes killing via apoptosis and cell cycle arrest in melanoma and human monoblastic leukemia (14, 17). It may also work in a novel apoptotic fashion that is Fas-, caspase-3, Bax, and Bak independent (23). Recently, there is evidence that WGA can kill via a completely different pathway. It has been demonstrated that WGA induces paraptosis-like cell death in cervical carcinoma cells (24). These different modes of killing, dependent on target cells, makes WGA an intriguing protein to study.

Because lectins, specifically wheat germ agglutinin, have been shown to be cytotoxic toward pancreatic cancer, osteosarcoma, hepatoma, etc. (19, 22), we screened three hematological malignancies [acute myeloid leukemia (AML), acute lymphoblastic leukemia (ALL), and non-Hodgkin lymphoma (NHL)] against a panel of lectins. AML is a common childhood leukemia. In patients who acquire the malignancy in adulthood it has a low survival rate (25). ALL is the most common pediatric cancer. If relapse occurs, patients have an even lower survival rate (26). NHL is an umbrella term for many different malignancies that originate in the lymphoid system (27). Because of this broad category, NHL is one of the most common cancers in the United States and the American Cancer Society estimates that more than 70,000 cases will be diagnosed in 2018 (28). Because these three cancer types are very common impacting large numbers of people, we looked at the cytotoxic effects of various lectins on these cancer cells.

## Methods

### Cell Culture

Human acute myeloid leukemia cell lines, OCI-AML3 and HL-60, obtained from DMSZ and American Type Culture collection, respectively, were cultured in sterile RPMI-1640 medium (R8758) with 10% Serum Plus II and 5% penicillin streptomycin. Human acute lymphoblastic leukemia cell lines, ALL-1 and ALL-2, were cultured in MEM medium (M4526) with 20% FBS. ALL-1 NSG cell line had been passaged through mice before freezing and usage. Human non-Hodgkin lymphoma cell lines, JVM2 and OCI-Ly10, were cultured in RPMI complete medium and in Iscove's DMEM (10-016CV) (20% SPII and 1% Glutamax), respectively. Non-cancerous control cells, HEK293 and OP9, were cultured in DMEM (sc-224478) and MEM, respectively. All cell lines were cultured at 37°C and 5% carbon dioxide. When cells reached confluency, they were passaged.

### Patient Samples

Primary patient AML cells were ordered from the Hematopoietic Stem Cell Core Facility at Case Western Reserve University and cultured in RPMI-1640 complete medium (10% FBS). Peripheral blood mononuclear cells (PBMCs), isolated from blood, were obtained from the Hematopoietic Stem Cell Core Facility at Case Western and cultured in RPMI-1640 complete medium (10% Serum Plus II). Human blood was also obtained from Hematopoietic Stem Cell Core Facility at Case Western Reserve University.

### Lectins

Lectins from: *Pisum sativum* (L5380), *Arachis hypogaea* (L0881), *Triticum vulgaris* (L9640), *Glycine max* (L1395), *Phaseolus vulgaris* (61764), *Agaricus bisprous* (L5640), *Lycopersicon esculentum* (L2886) were purchased from Sigma-Aldrich, dissolved in sterile phosphate-buffered saline (PBS), and stored at 4°C in a concentration of 1 mg/mL. Succinyl-WGA (W0110) and wheat germ agglutinin FITC-conjugate (L4895), were purchased at Vector Laboratories and Sigma-Aldrich, respectively. These variants were also dissolved in sterile phosphate-buffered saline (PBS) and stored at 4°C in a concentration of 1 mg/mL. Lectin from *Sambucus nigra* (ZB0106) was purchased from Vector Laboratories. Detailed information on each lectin is included in Table 1 and obtained from Sigma-Aldrich product sheets.

**Table 1:** All lectins used and their name, source, molecular weight, and sugar specificities.

**Lectin**	**Source^a^**	**Molecular weight^b^ (kDa)**	**Sugar specificity^c^**
Wheat germ agglutinin (WGA)	*T. vulgaris* (wheat)	36	(GlcNAc)_2_ & NeuNAc
Succinyl-Wheat germ agglutinin (sWGA)	*T. vulgaris* (wheat)	36	(GlcNAc)_2_
Pisum sativum agglutinin (PSA)	*P. sativum(pea)*	49	α-man
Peanut agglutinin (PNA)	*A. hypogaea* (peanut)	120	Gal-β(1 → 3)-GalNAc
Soybean agglutinin (SBA)	*Glycine max* (soy)	110	GalNAc
Phytohemagglutinin (PHA)	*P. vulgaris* (red kidney bean)	126/128	Oligosaccharide
Agaricus bisporus lectin (ABL)	*A. bisporus* (mushroom)	58.5	β-gal(1 → 3)GalNAc
Lycopersicon esculentum lectin (LEL)	*L. esculentum* (tomato)	71	(GlcNAc)_3_
Sambucus nigra lectin (SNA)	*S. nigra* (elderberry)	140	αNeuNAc(2 → 6)gal & GalNAc

*Information obtained from Sigma-Aldrich data sheets*.

a, b, c *All values and specificities from Sigma Aldrich product information sheet*.

### Reagents

Neuraminidase (N7885) was purchased from Sigma-Aldrich and stored at 4°C. Propidium Iodide/RNAase staining kit (P40875) was obtained from Cell Signaling Technology. Annexin V Apoptosis Detection Kit with PI (640914) was purchased from BioLegend. Trypan blue (T8154) was purchase from Sigma-Aldrich. Alsever's Solution was prepared using Sally E. Grimes's protocol (29). Citric acid (1940) and Sodium chloride (BP-358-10) was purchased from Sigma-Aldrich, while Sodium citrate (S-4641) and D-glucose (G-5767) were purchased from Fisher Scientific.

### Cell Death Assay

HL60 and OCI cells were seeded in 12-well plates at a concentration of 250,000 cells/mL (1 mL per well). Cells were treated with WGA at various concentrations on day 0, then again at 24 h intervals up until the final day of measurement. Two microliter PBS were added as a negative control. Cell count and cell viability were assessed using 1:1 trypan blue staining (Sigma-Aldrich) and an automated cell counter (Bio-Rad TC-20). Measurements were conducted in triplicate. Data was graphed and analyzed using GraphPad Prism 7.

### Apoptosis Assay

Cells were treated with 2 μg/mL WGA for 24 h. Cells were centrifuged at 300 × *g* for 5 min and the supernatant was removed. The pellet was washed with PBS and resuspended in 100 μL Annexin V/ Propidium iodide (AV/PI) buffer. Samples and positive controls were incubated with 3 μL of Annexin V antibody and 10 μL of Propidium Iodide for 15 min at room temperature. The samples were run using fluorescence-activated cell sorting (FACS BD Accuri™C6). 20,000 events were recorded per sample. AV/PI kit from Biolegend, USA was used to perform apoptosis assay.

### Cell Cycle Analysis

Cells were seeded at 250,000 cells per mL in 4 mL and treated with WGA. Cells were spun at 600 rpm for 5 min and washed with PBS twice. Pellet was resuspended in PBS and vortexed to make single cell suspension. While vortexing the sample, 1 mL of ice-cold 70% ethanol was added. Samples were incubated overnight in −20°C. Then, samples were pelleted, washed, resuspended in PBS, and incubated with 100 μL of Propidium Iodide at room temperature for 15 min. Samples were analyzed with FACS, counting 10,000 events. Events collected were gated on live cell populations, avoiding debris and aggregate populations.

For cell aggregation/agglutination assay, HL-60, OCI, and healthy human white blood cells (WBCs) were seeded in 12-well plates at a concentration of 250,000 cells/mL (1 mL per well). Cells were treated with either 2 μg/mL WGA or with 2 μL PBS as a negative control. After 20 h treatment, cells were assessed at 10x magnification using bright field microscopy (Leica DM IL LED) and captured using Leica LAS X imaging software.

### WGA Binding

WGA-FITC working stock was made by diluting the 1 μg/mL stock solution. HL-60 AML cells were seeded at 250,000 cells per mL and treated with 0.5 μg/mL WGA-FITC at 37°C. At each time point, samples were washed with PBS and analyzed using FACS.

### Sialic Acid-Based Treatments

Cells were treated with succinylated-WGA (sWGA) at 2 μg/mL at 37°C for 24 h. Samples were counted using trypan blue. For neuraminidase pre-treatment, the protocol described in Schwarz et al. where 4 million cells in 2 mL serum free media are incubated with 50 mU/mL neuraminidase for 1 h at 37°C was used (22). Samples were washed twice in complete media and seeded in wells at 250,000 cells/mL. Samples were treated with WGA in the same manner as described above. Cells were stained with Propidium iodide and cell viability was determined using flow cytometry.

### E-670 Cell Proliferation Assays

OCI AML-3 and HL-60 cell lines were labeled with 1 mM cell proliferation Dye eFluor 670™ (Thermo Fisher Scientific) as per manufacturer's instructions. After staining cells were washed two times and cultured at 37°C in media alone or in the presence of 2.5 μg/mL WGA for the indicated times. Proliferation of live cells was assessed via flow cytometry (Accuri 6C).

### *In vitro* Toxicity

Two AML patient samples were treated in the absence or presence of with 2 μg/mL WGA for 24 h at 37°C. The samples were analyzed for viability by flow cytometry. OP9 and HEK293 cells were plated and incubated for 24 h with doses of WGA. Confocal images were acquired using EVOS® XL Core Imaging System.

### Hemagglutination (HA) Assay

The protocol designed by Virapur®was modified as follows (30). Acquired mouse blood was stored in prepared Alsever's solution. After three washes in PBS, 10% blood stock solution was made in PBS. A working stock (5%) solution was made using the 10% stock and PBS. A serial dilution of WGA (50 μg to 0.09 μg/mL) was prepared using a round-bottomed 96-well plate. 0.0 μg/mL WGA was used as a negative control. The plate was incubated for 30–60 min at room temperature and images were taken. The plates were analyzed by looking for “buttons” in each well. Diffuse blood in the well is analyzed as hemagglutination. Experiments were performed on human blood, as well, but the blood was not stored in Alsever's solution because it already contains the anti-coagulant heparin.

### *In vivo* Toxicity

Twelve-week-old C57BL mice were given WGA (2 mg/kg) by intraperitoneal injection on days 1, 4, and 8. Mouse weights were also taken throughout the time of administration. After WGA administration was completed, the mice were sacrificed and spleen, kidney, and liver were harvested and fixed in formalin. H&E staining was completed at the Immunohistochemistry Core Facility at CWRU. Blood was collected in EDTA-coated tubes and analyzed using HemaVet.

### Xenograft *in vivo* Model

NSG mice were subcutaneously injected with 5 × 10^6^ HL-60 cells to generate solid AML xenograft mice model, followed by three intra-tumor injection of WGA or PBS.

### Statistical Analysis

Data were analyzed using unpaired Student's *t*-test. All experiments were done in triplicate (*n* = 3). *P*-values in figures correspond to: ns = non-significant (>0.05), ^*^P < 0.05, ^**^P < 0.01, ^***^P < 0.001. All graphs were made and statistical analyses were performed using GraphPad Prism program.

## Results

### Lectins Demonstrate Variable Cytotoxic Activity Toward Different Cancers

**Figure 1:**
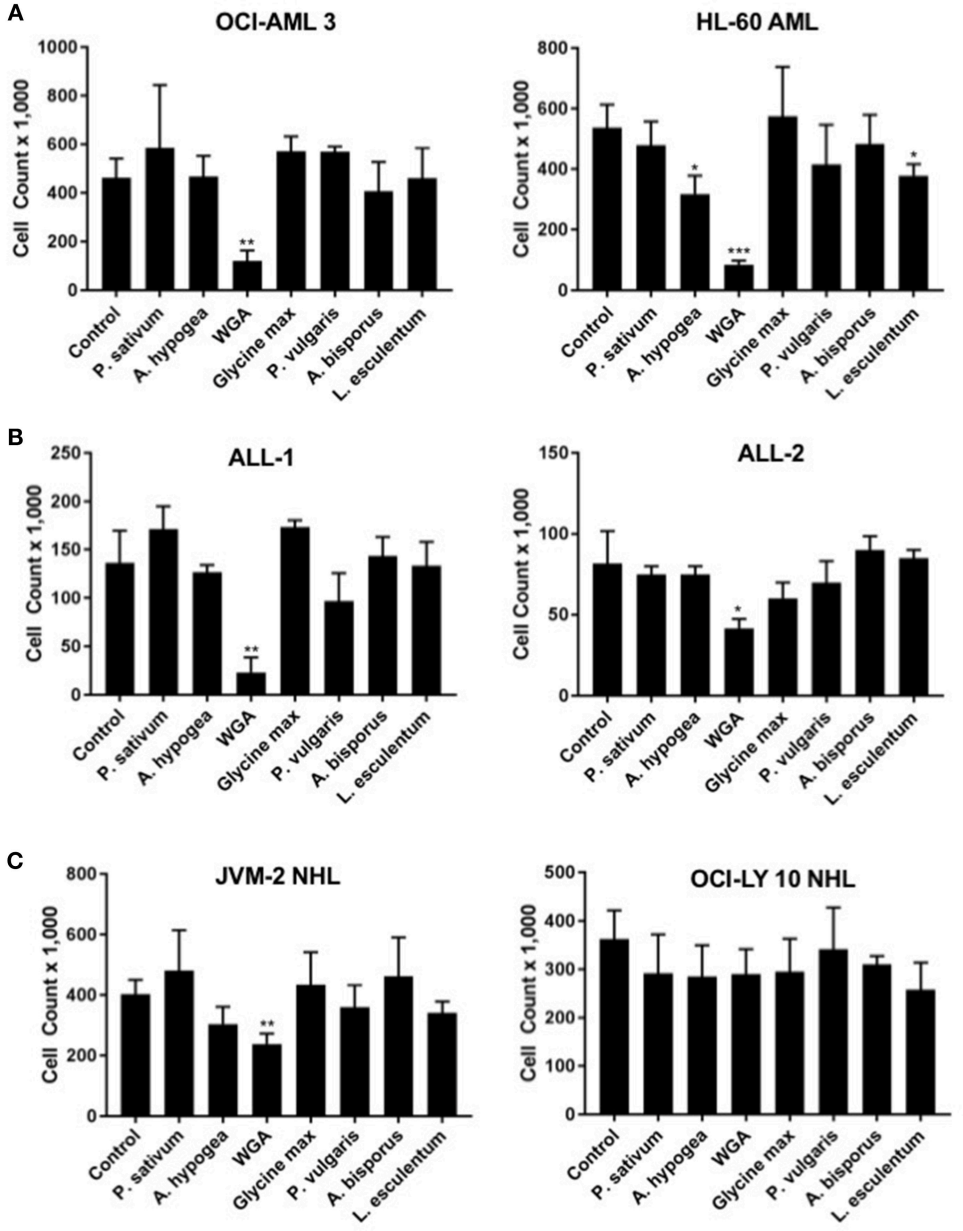
Lectins demonstrate variable toxicities to cancer types. **(A)** Viable cell count of AML (OCI-AML3 and HL-60) cells treated with different dietary lectins as indicated. **(B)** Viable cell count of ALL (ALL-1 and ALL-2) cells treated with different dietary lectins as indicated. **(C)** Viable cell count of NHL (JVM2 and OCI-Ly10) cells treated with different dietary lectins as indicated. All seven lectins administered at 2 μg/mL for 24 h for all cell types. Un-labeled bars were non-significant compared to control. ^*^p < 0.05, ^**^p < 0.01, ^***^p < 0.001, and ns > 0.05.

In order to determine how WGA killing compares to other lectin treatment, we looked at a panel of varied lectins. Cytotoxic effects of seven different dietary lectins at 2.0 μg/mL were tested toward AML, ALL, and NHL. Two cell lines from each disease type were used. Wheat germ agglutinin (WGA) consistently showed significant cytotoxicity toward all five cancer cell lines, except OCI-Ly10. As shown in Figure 1, WGA-mediated cell killing of OCI-AML3 (*p* = 0.0028), HL60 (*p* = 0.0005), ALL-1 (*p* = 0.0058), ALL-2 (*p* = 0.03), and JVM2 (*p* = 0.009) were statistically significant (Figures 1A–C). All other lectins tested did not show significant cytotoxic activity toward these cancer cells. Binding specificities of all these lectins are detailed in Table 1.

### WGA Binds and Kills Cancer Cells in a Dose- and Time-Dependent Manner

**Figure 2:**
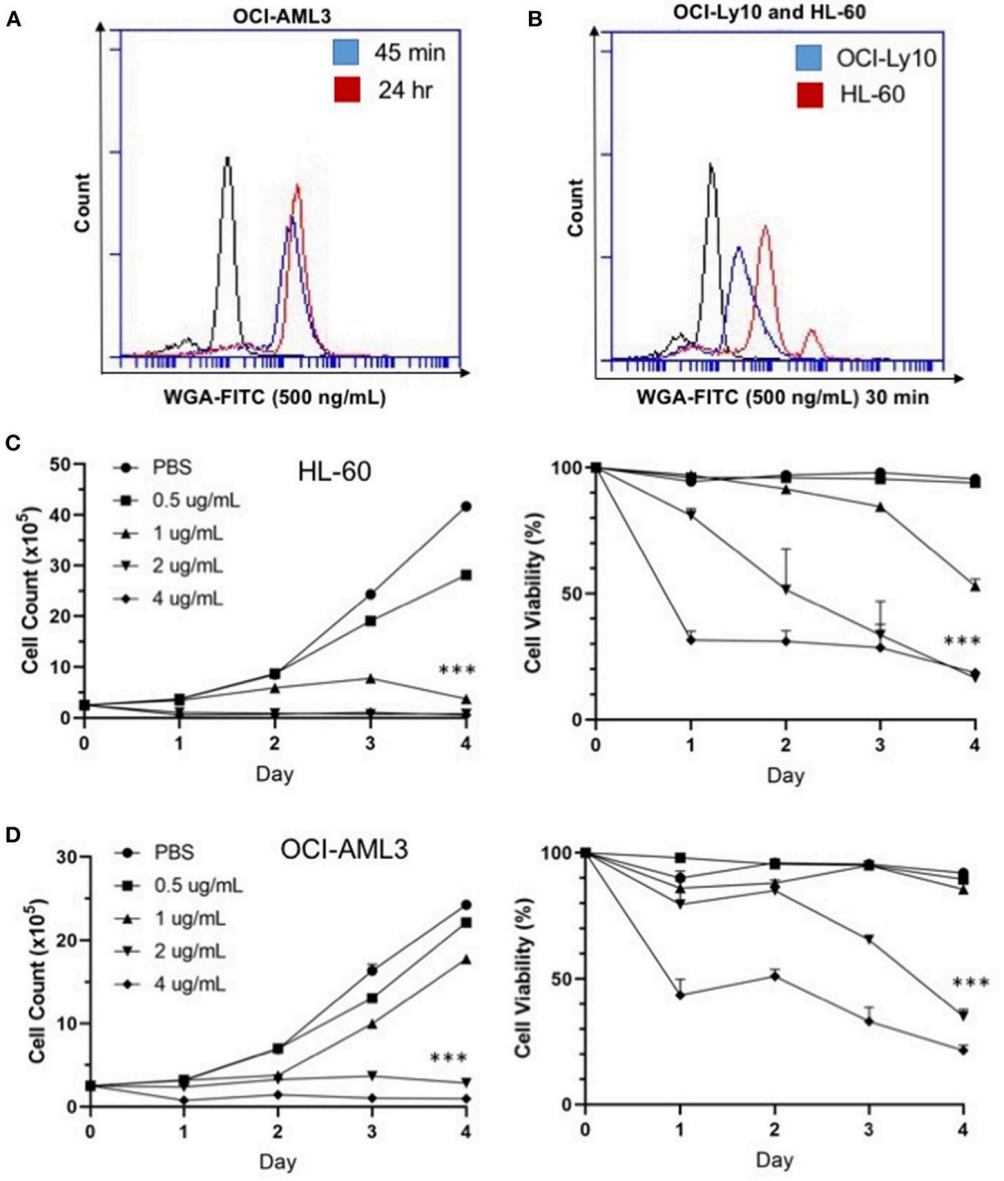
WGA binds and kills in a dose- and time-dependent manner. **(A)** OCI-AML3 cells treated with WGA-FITC (500 ng/mL) and analyzed for binding using flow cytometry. Binding to cells at 45 min (blue peak) and 24 h (red peak) compared to control (black peak). **(B)** OCI-Ly10 and HL-60 cells treated with WGA-FITC (500 ng/mL) for 30 min and analyzed for binding using flow cytometry. **(C,D)** Viable cell count and percent viability of HL-60 **(C)** and **(D)** OCI-AML3 with WGA treatment (0.5, 1.0, 2.0, and 4.0 μg/mL) for 1–4 days, counted using trypan blue. ^***^p < 0.001.

We were interested at which dose and time WGA would be most effective, so we looked at binding and killing at different doses and time points. We utilized a FITC-labeled WGA at 0.5 μg/mL, in order to analyze cellular binding using flow cytometry at a sub-lethal WGA dose. From the flow cytometry data, it is evident that within 45 min of incubation with WGA, the lectin is bound to the surface of the OCI-AML3 cells. This binding is present up to 24-h after incubation (Figure 2A). We also wanted to elucidate the relationship between binding and killing, so we looked at binding of WGA to OCI-Ly10 compared to HL-60 AML. We show that there is a significant reduction in WGA-binding to OCI-Ly10 (Figure 2B). This reduction in binding coincides with the absence of WGA-induced cell killing of OCI-Ly10 (Figure 1C).

Sensitivity of AML cells to WGA up to 4 days was calculated using OCI-AML3 and HL-60 cell lines, using four different doses. Significant killing for HL-60, occurred at 1.0, 2.0, and 4.0 μg/mL, starting from day 1 of WGA treatment (Figure 2C). We analyzed cell killing at day 1, 2, 3, and 4. At day 4, almost all cells were killed except for 0.5 μg/mL WGA treated wells. Viable cell count data shows that most of the cells were killed at day 1 itself. Dose kinetics of OCI-AML3 cells show significant killing at 2.0 and 4.0 μg/ml WGA (Figure 2D). HL-60 was more sensitive to WGA induced cell death, even at 1.0 μg/ml, while OCI-AML3 was sensitive to 2.0 μg/ml WGA.

### WGA Kills Different Subtypes of Primary Patient AML Cells

**Figure 3:**
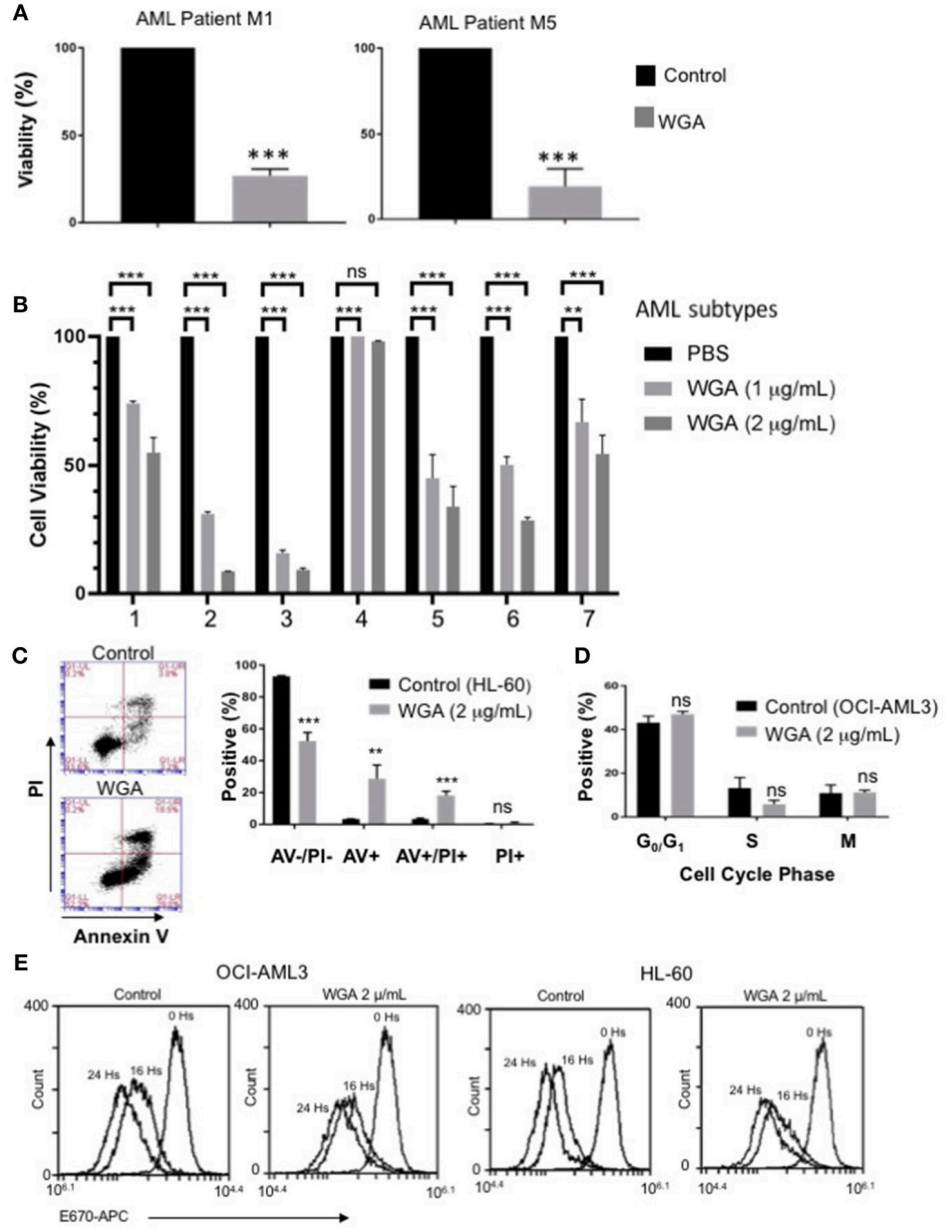
WGA is toxic to primary patient cells and kills AML cells without altering cell cycle. **(A,B)** Percent viability calculated using FSC/SSC live gating of primary AML patient cells belonging to different subtypes **(A)** M1&M5 **(B)** M4Eo; inv16 (1), AML with MDS related changes (2), M2 with MDS-related changes (3), M2(4), CEBPA;c-Kit;TET2(5), NPM1;IDH1(6), IDH1;DNMT3a;FLT3-TKD;trisomy8(7) all treated with or without 2 μg/mL WGA for 24 h. Control was standardized to one hundred percent. **(C)** Annexin V/Propidium Iodide staining of HL-60 cells treated with WGA (2.0 μg/mL) for 24 h. **(D)** Cell cycle analysis of gated live OCI-AML3 cells treated with WGA (2 μg/mL) for 24 h. **(E)** E-670 cell proliferation assays using OCI-AML-3 and HL-60 cell lines in media alone or in the presence of 2.5 μg/mL WGA for the indicated times. Proliferation of live cells was assessed via flow cytometry (Accuri 6C). ^**^p < 0.01, ^***^p < 0.001, and ns > 0.05.

In order to further evaluate our findings using AML cell lines, we tested if WGA has same effect on primary cells derived from AML patients. AML can be divided into eight different subclasses (M0-M7) based on the differentiation status, according to the French-American-British (FAB) classification (31). AML also can be divided into subtypes based on WHO classification of genetic abnormalities (32). Primary acute myeloid leukemia blood samples from two AML patients (subtype M1 and M5) were treated with 2 μg/mL WGA for 24 h and analyzed by flow cytometry. The flow cytometry count of viable cells (as determined by analyzing forward and side scatter) demonstrates very significant cell killing at this dose of WGA for both patient samples (*p* = 0.0001) (Figure 3A). Further, we analyzed sensitivity of seven more different AML subtypes to WGA induced killing and found that all six subtypes except M2 subtype showed significant cell killing after exposure to 1.0 and 2.0 μg/ml WGA (Figure 3B). M2 subtype with MDS related changes showed maximum killing, even after exposure to 1.0 μg/ml WGA (Figure 3B).

After confirming WGA induced cell death in different AML cell lines and patient cells, we wanted to elucidate the specific method of cell killing that WGA utilizes toward AML cells by focusing on cell death and cell cycle. Annexin V (AV)/Propidium Iodide (PI) stain can be used to distinguish between necrotic and apoptotic cell death. AV staining works by binding to phosphatidylserine, which normally resides on the inner cell membrane. However, in early apoptosis, the cellular membrane undergoes changes where phosphatidylserine is present on the outer membrane. PI staining works due to cell membrane rupture, which allows the stain to enter the cell which are in late apoptotic phase or undergoing necrotic death. Flow cytometry scatter demonstrates that at 2 μg/mL there are AV+ and PI+ cells. There is a significant difference between AV–/PI–, AV+, and AV+/PI+ of control and treated HL-60 AML cells (*p* = 0.0002, *p* = 0.0068, *p* = 0.0006). However, there was no statistical difference between PI+ (alone) of control and treated (Figure 3C). There is also a significant difference between control and treated cells if all positively staining populations (AV+, AV+/PI+, PI+) are grouped together (*p* = 0.0002). Because it has been shown in the literature that WGA can disrupt cell cycle (14, 17), we tested AML cell cycle changes after being incubated with WGA. Fixing cells and staining with PI allows for the different phases of the cell cycle to be distinguished. Flow cytometry analysis shows that OCI-AML3 cells incubated with 2 μg/mL WGA for 24 h have non-significant changes to G_0_/G_1_, S, and G_2_/M phases compared to untreated control cells (Figure 3D). In order to analyze the effect of WGA on cell proliferation, we performed E-670 cell proliferation analysis of OCI-AML3 and HL-60 cells before and after 16 and 24 h of WGA treatment (Figure 3E). We did not see any significant changes in staining of these cells. Since WGA induced AML cell killing is a rapid process happening within 24 h, we could not analyze further time points.

### WGA Induced AML Cell Death Depends on Both Interaction With Sialic Acid and GlcNAc Binding

**Figure 4:**
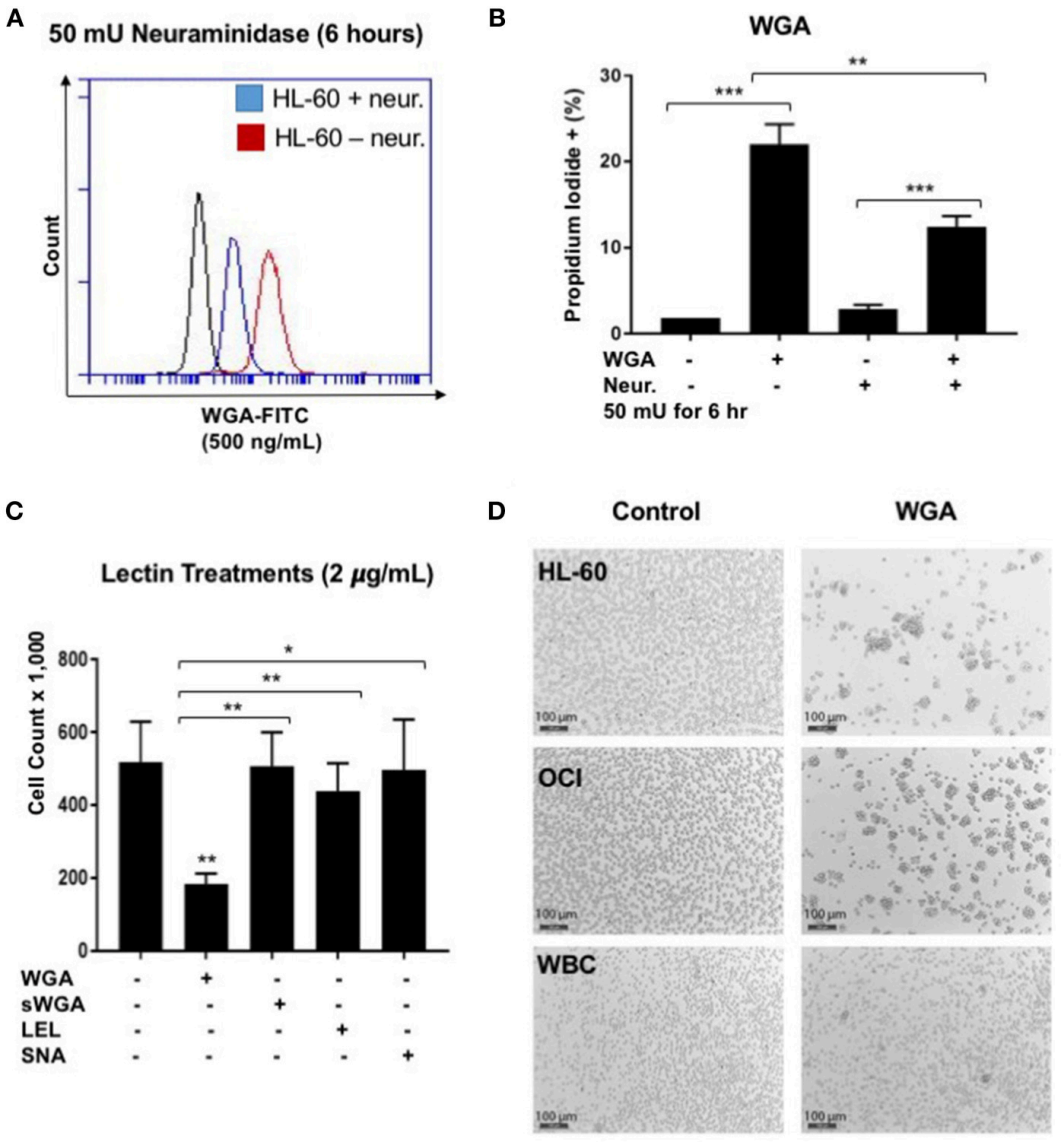
WGA effect on AML is sialic acid dependent. **(A)** HL-60 cells treated with WGA-FITC (500 ng/mL) to analyze WGA binding using flow cytometry. Cells untreated with neuraminidase (red peak) were overlaid with cells treated with neuraminidase (50 mU/mL) for 6 h. **(B)** Percentage Propidium iodide positive HL-60 cells treated with WGA (4 μg/mL) for 4 h were analyzed with flow cytometry. HL-60 cells were either untreated or pre-treated with neuraminidase (50 mU/mL) for 2 h. **(C)** Viable cell count of OCI-AML3 cells treated with 2 μg/mL WGA, sWGA, LEL, and SNA for 24 h counted using trypan blue. **(D)** HL-60, OCI, and healthy human white blood cells (WBCs) treated with either 2 μg/mL WGA or with 2 μL PBS as a negative control and after 20 h treatment, cells were assessed at 10× magnification using bright field microscopy. Scale bar 100 μm shown. ^*^p < 0.05, ^**^p < 0.01, ^***^p < 0.001, and ns > 0.05.

WGA binds primarily to GlcNAc and also interacts with sialic-acid containing glyconjugates and oligosaccharides. We tested which binding activity of WGA contributes to its cancer killing activity. Neuraminidase, also called sialidase, is an enzyme that can catalyze the hydrolysis of sialic acid glycosidic linkages. After neuraminidase pre-treatment, the sialic acid should be cleaved off the cell membrane. Hence, to determine the role of sialic acid interaction with WGA in WGA-mediated cancer cytotoxicity, we pre-treated HL-60 cells with neuraminidase (50 mU/mL) for 2 h and then incubated with 4 μg/mL WGA for 4 h. After treatment, we stained the cells with Propidium iodide and analyzed using flow cytometry. There was a significant increase in PI staining in the WGA-treated groups with and without neuraminidase (*p* = 0.0001, *p* = 0.0002, respectively) (Figure 4B). However, when the cells were pre-treated with neuraminidase followed by WGA treatment, the amount of PI staining is significantly reduced compared to cells treated with WGA alone (*p* = 0.0033) (Figure 4B). We used FITC-labeled WGA at sub-toxic levels (500 ng/mL) analyzed with flow cytometry to confirm that neuraminidase reduced WGA binding. At 6 h, there was a noticeable reduction in binding to HL-60 cells (Figure 4A).

We also wanted to look at other lectins, specific for the carbohydrate moieties WGA interacts with. Succinyl-WGA is a modified form of WGA that only binds GlcNAc. We used succinyl-WGA to determine the role of GlcNAc binding in WGA cytotoxicity. We found a significant difference between OCI-AML3 cells treated with succinylated-WGA and unmodified WGA (*p* = 0.0237) (Figure 4C), showing that the sialic acid interaction is important for WGA-induced killing. Cells treated with 2 μg/mL SNA lectin, which are specific for sialic acid attached to terminal galactose in α-2,6 and to a lesser degree α-2,3 linkage, are not affected compared to control (Figure 4C). OCI-AML3 cells treated with 2 μg/mL LEL, which binds GlcNAc, are also not affected compared to control (Figure 4C); however, HL-60 cells treated with LEL showed a significant decrease (*p* = 0.0302) (Figure 1A). We also observed cell aggregation/agglutination in HL-60 and OCI-AML3 cells preceding cell death (Figure 4D). WGA did not agglutinate normal white blood cells (WBC's) at this concentration and time point (Figure 4D).

### WGA Exhibits Limited Toxicity to Normal Cells *in vitro* and *in vivo*

**Figure 5:**
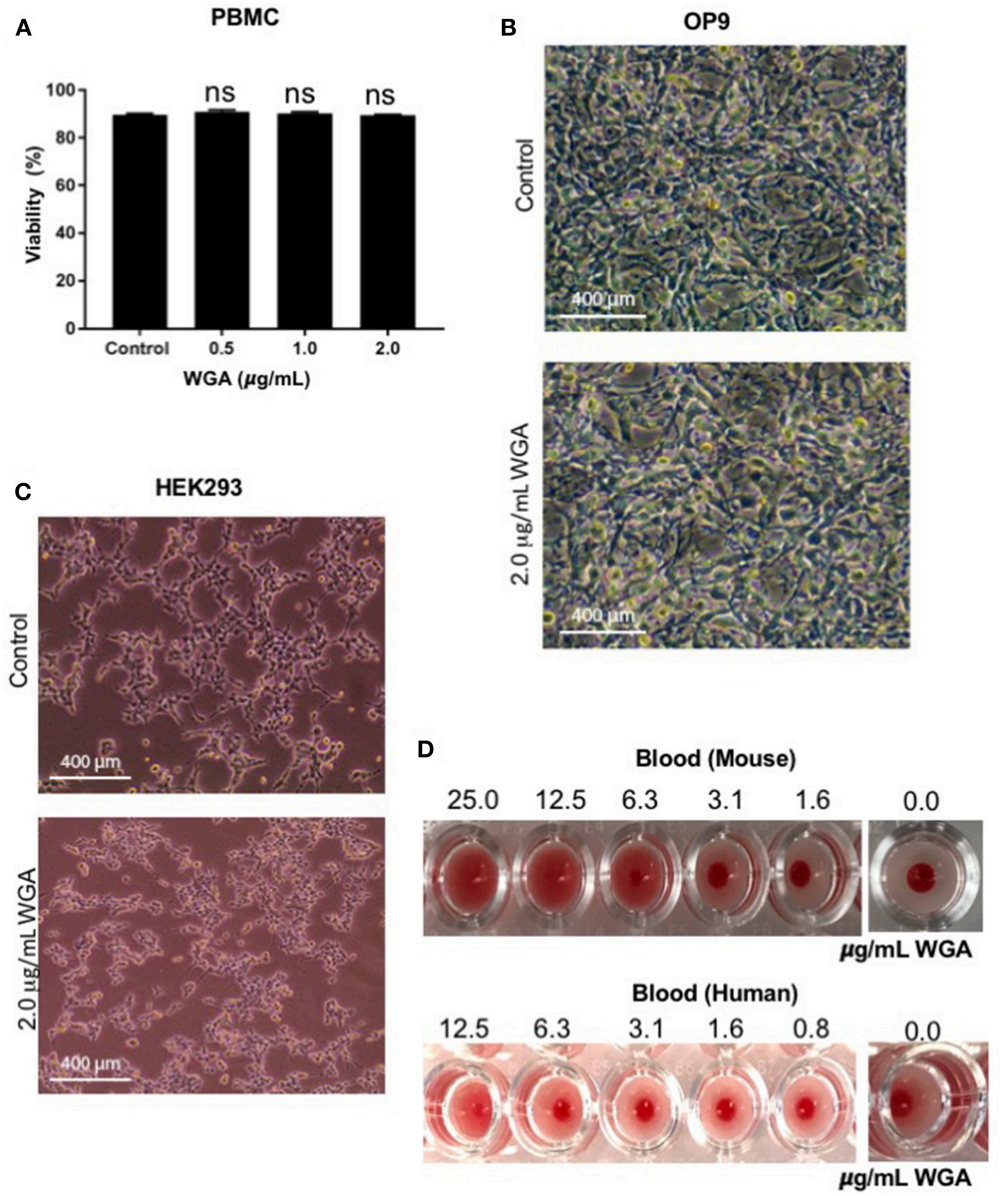
WGA treatment exhibits little or no toxicity toward normal cells *in vitro*. **(A)** Viable peripheral blood mononuclear cells (PBMCs) treated with 0.5, 1.0, and 2.0 μg/mL WGA for 24 h were calculated by PI uptake using flow cytometry. **(B)** Light microscopy of OP9 stromal cells showing phenotype. Cells were treated with 2.0 μg/mL WGA for 24 h and imaged. Scale bar 400 mm shown. **(C)** Light microscopy of HEK293 cells showing phenotype. Cells were treated with 2.0 μg/mL WGA for 24 h and imaged. Scale bar 400 mm shown. **(D)** Hemagglutination assay on 96-well micro-plate of mouse and human blood using serial dilution of WGA (25 to 1.6 μg and 12.5 to 0.8 μg, respectively). Absence of lectin control (0.0 μg) for mouse and human blood are included. ns > 0.05.

At this point, we had demonstrated WGA kills leukemia cells at 1.0–2.0 μg/mL, but we could not discount indiscriminate killing. Because of this concern, we tested various non-cancerous cells with WGA. Propidium iodide staining and flow cytometry analysis shows no significant changes between peripheral blood mononuclear cells treated without WGA and with 0.5, 1.0, and 2.0 μg/mL WGA (Figure 5A). OP9, a stromal cell line, was treated with 2.0 μg/mL WGA and no significant morphological changes were apparent microscopically. The cells were not detached from the plates and maintained normal shape (Figure 5B). HEK293 cells were also treated with 2.0 μg/mL WGA and imaged. There were no morphological changes after incubation (Figure 5C). All these data points to the different toxicity of WGA toward cancer cells and normal cells. Because WGA is known to cause red blood cell (RBC) agglutination (20), we wanted to test whether the WGA dose we are using for the cytotoxic assay causes agglutination in mouse and human RBCs. Hemagglutination assays of human and murine blood after exposure to WGA demonstrated lack of hemagglutination at the indicated doses used. This is evident by the button of blood settled to the bottom of the well. A positive hemagglutination result is diffuse blood in the well as shown in the higher doses imaged (Figure 5D).

**Figure 6:**
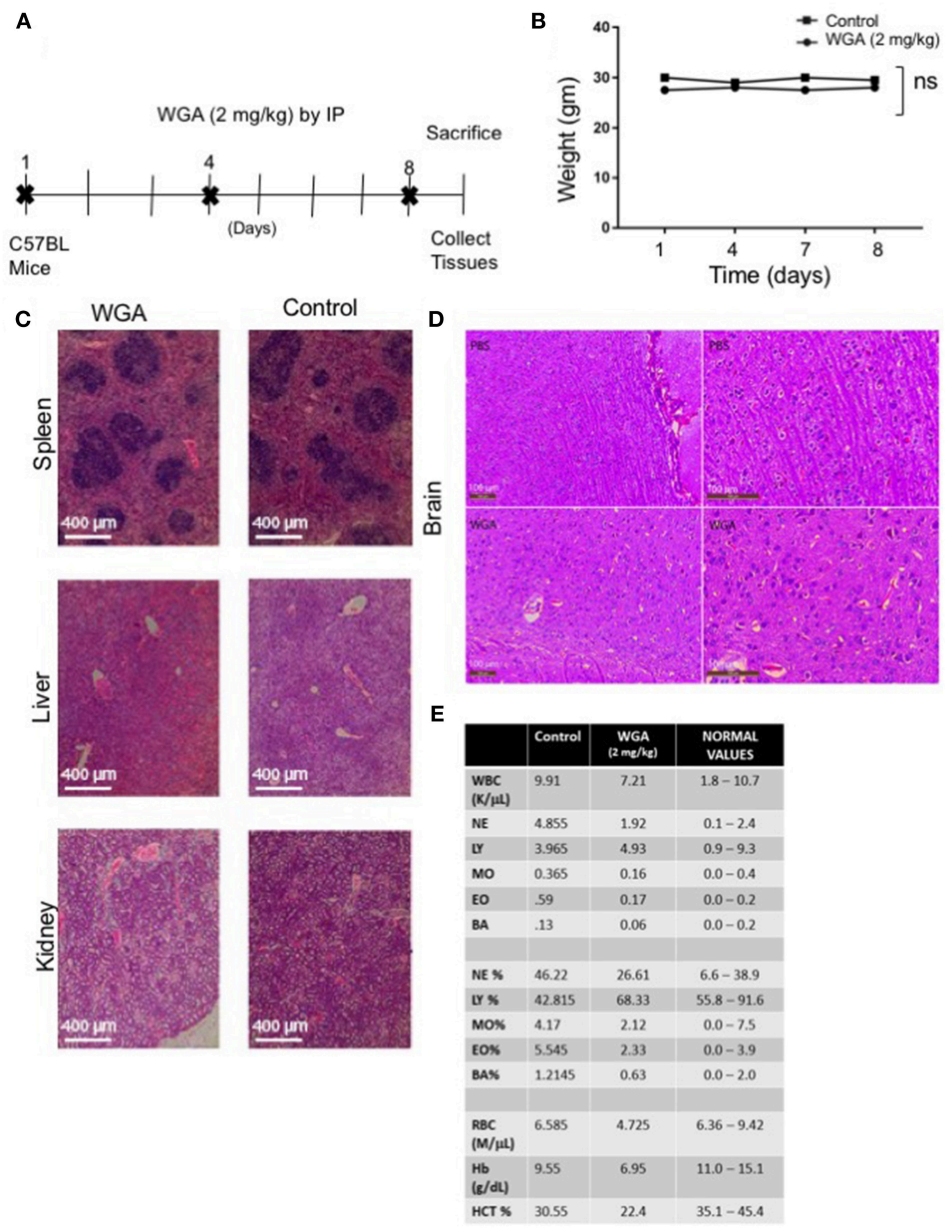
WGA treatment demonstrated little toxicity to normal cells *in vivo*. **(A)** Treatment scheme where 2 mg/kg WGA was IP administered to two mice on days 1, 4, and 8. Mice were sacrificed on day 9. **(B)** Weights of mice treated with WGA during duration of treatment. **(C)** Histological analysis of WGA-treated tissues. Spleen, liver, and kidney stained with H&E and imaged by light microscopy at 10X magnification. Scale bar 400 μm shown **(D)** Histological analysis of Hematoxylin&Eosin stained brain tissues from PBS and WGA (5 mg/kg) injected mice. Scale bar 100 μm shown **(E)** HEMAVET blood toxicity analysis of treated mice after sacrifice compared to control mice and normal values. Normal values were given with HemaVet instructions. ns > 0.05.

Finally, because we had determined effect of WGA on normal cells *in vitro*, we tested whether WGA is toxic *in vivo*. We conducted a study to obtain information of WGA dose toxicity where WGA was injected (2 mg/kg) by IP to 2 C57BL/6 mice on days 1, 4, and 8. Mice were sacrificed on day 9 for further analysis (Figure 6A). Age and sex-matched, non-treated mice served as controls. The mortality and changes to body weight, clinical signs, gross observation, organ weight, and histopathology of principal organs (spleen, liver and kidney) were monitored. We found no mortalities, WGA treatment-related clinical signs, changes to the body and organ weights, or gross and histo-pathological findings (Figures 6B,C). Since some reports say WGA can cross blood brain barrier (BBB) (33), we analyzed brain tissue from WGA (5 mg/kg) and PBS injected C57BL/6 mice. Histochemical stainings of brain sections showed normal structures comparable to PBS injected mice, with no signs of toxicity (Figure 6D).

We also analyzed different blood cells using HEMAVET. WGA-treated mice displayed cell counts within normal ranges as shown in Figure 6E, except for slightly reduced red blood cell count values, such as RBC, hemoglobin, and hematocrit levels (Figure 6E). These suggest that WGA at this dose is safe to use *in vivo*.

### WGA Induced AML Cell Killing in Xenograft Mouse Model

**Figure 7:**
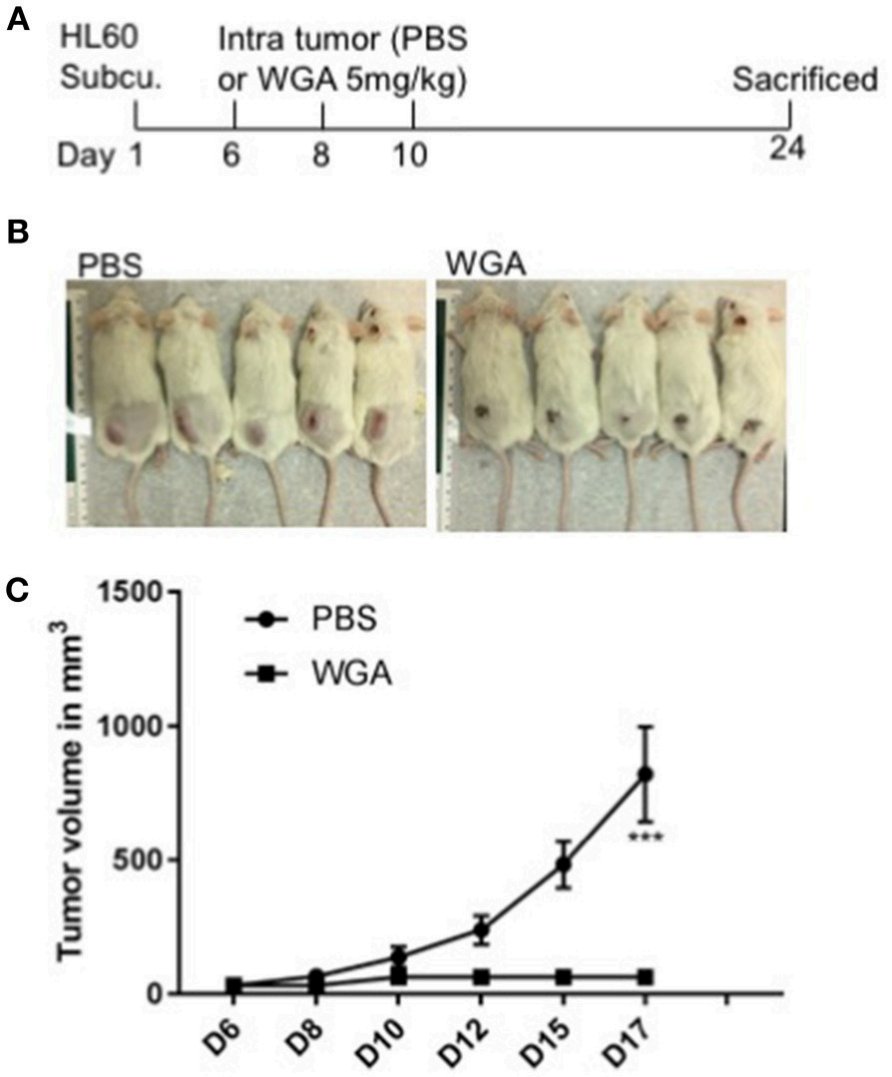
WGA induced AML cell killing in xenograft mouse model. **(A)** Treatment scheme- HL-60 cells injected at day 1 and at day 6, 8 and 10 WGA 5mg/kg injected intratumorally and mice were sacrificed at day 24. **(B)** Pictures of mice treated with PBS or WGA **(C)** Graph showing tumor volume (mm^3^) of PBS and WGA injected mice. ^***^p < 0.001.

We further evaluated WGA killing of AML cells *in vivo* using a xenograft mouse model. Severely immunodeficient NSG mice were used for this study. HL-60 AML cells were injected subcutaneously (s.c.) into NSG mice and injected WGA intra-tumorally at days 6, 8, and 10 (Figure 7A). Mice tumor volume was measured every alternate day and WGA injected mice showed a very significant inhibition in tumor progression, compared to PBS injected mice (Figures 7B,C). We sacrificed these mice at day 24. Tumors in PBS injected mice reached volume upto 1,000 mm^3^, while there were no measurable tumors in WGA injected mice. NSG mice lack mature lymphocytes including B cells, T cells and NK cells, so it is highly likely that AML cell killing by WGA lectin seen in this model is a direct anti-leukemic effect by WGA. Mice injected with WGA did not show any obvious signs of toxicity suggesting that this therapeutic strategy may be safe, and it is worthy of further development for AML, provided route of administration is optimized.

## Discussion

Dietary lectins, particularly wheat germ agglutinin, have been shown to have important anti-cancer properties (18, 19, 22). However, knowledge of lectins' impact on hematological malignancies, such as AML, ALL, and NHL, is lacking. Because these malignancies are in need of potential new treatments, exploring dietary lectins can be a valuable starting point. The panel of lectins chosen encompasses various carbohydrate binding specifities and sizes, as well as sources of origin. We demonstrated that lectins' effects on cancer is variable, with the vast majority of dietary lectins having no impact at all on cancer cell viability. WGA showed significant cell killing against five cell lines tested (out of six). WGA was ineffective against OCI-Ly10. Normal cells were also insensitive to doses of WGA, where it showed significant killing activity against cancer cells. High concentrations of WGA will kill normal cells as well, so choosing the right dose of WGA is key to the success of treatment. We demonstrate the dose specificity of WGA with AML cells showing a significant cytotoxic effect on AML cells but not with the normal cells. WGA killed all AML subtypes tested except M2, this has to be tested further using many M2 patient samples and if WGA fails to kill this particular subtype, it has to be studied further. The exact reasons for this specificity is not known. WGA binds to GlcNAc and it also interacts with sialic-acid containing glyconjugates and oligosaccharides. Since most of the cancer cells are hyper O-GlcNAcylated and hyper silalylated (34–39), we could speculate that it might be the differences in levels of GlcNAc expression and presence of sialylation on cell membrane in different cells which accounts for WGA's cell binding and toxicity specificity. At higher doses, the mechanism of cell killing may be agglutination of cell membrane.

Our data and the literature show the importance of both sialic acid and GlcNAc in the cytotoxicity of WGA. However, the role of each carbohydrate moiety in the lectin-induced death of cells might vary from cell to cell. Sialic acid-specific lectins and GlcNAc-specific lectins were not able to kill AML cells on their own, signifying both properties are necessary for cell killing. If sialic acid is removed using neuraminidase, there is a reduction in binding and killing. The dual interaction to both carbohydrate moieties might be required because of the different locations of the carbohydrates within the cells. The sialic acid interaction may occur outside the cell on the cell membrane, while the GlcNAc binding may occur within the cell after internalization as proposed by Schwarz et al. (22). However, this mechanism of action may be specific to the pancreatic cancer cells used in the study. Further studies are required to understand any AML cell specific mechanism. Sialic acid and GlcNAc interaction within the cell upon internalization may also be key to the cell killing effects. These cell specific differences could also be why the method of killing varies by cancer type. Mechanisms of cytotoxicity by WGA includes apoptosis, necrosis, paraptosis, and cell cycle arrest. Our data demonstrates WGA induces apoptosis and necrosis, but cell cycle analysis revealed no significant differences.

WGA injected into the tumor arrested tumor growth in an NSG xenograft AML model. PBS injected mice had large tumors as expected, which excludes the possibility that intratumoral injection procedure has any effect on tumor growth. Since we used severely immune-compromised mice which lacks a proper innate immune response, the AML killing effect observed might be solely from WGA's direct effect on AML. Studies in the past suggested the possibility that WGA has harmful effects, however, several recent studies have re-evaluated many of those assumptions and suggested that WGA dangers are either non-existent or have limited effects (40, 41). Importantly, the *in vitro* and *in vivo* concentrations of WGA used in this study is very low and no toxicity is reported using this low concentration. Interestingly, more recently dietary lectins including WGA have been associated with the beneficial effect on health, including reduced risks of type 2 diabetes, cardiovascular disease, some types of cancer and weight management (41). Chronic exposure of high doses of WGA can lead to toxic effects like development of anti WGA antibodies, platelet aggregation or red blood cell (RBC) agglutination. We used low doses of WGA and short exposure timings, where these kind of toxicity is not a concern. WGA has been shown to elicit pro-inflammatory conditions, and its toxic effects could only be seen at a very high dose (of 7 g/kg body weight over a period of 10 days) in the normal gastrointestinal tract of rats, suggesting that WGA being non-toxic in a huge range (21, 40). A final word on toxicity or atoxicity of WGA is pending due to lack of *in vivo* studies, whereas microgram range of WGA used for targeting or carrier system is unlikely to provoke toxic effects (40).

The current therapies for treatment of AML include chemotherapy, radiation therapy, and stem cell transplant. These therapies rely on cell killing and differentiation which lead to cell death. AML treatment regimen can also change depending on the age and health of the patient. In a young patient, induction therapy of high doses of cytarabine and daunorubicin will be used to clear as much of the tumor burden as possible. Once the tumor is cleared, lower doses of these drugs will be used for maintenance. In older and unhealthy patients, these high doses are contraindicated because of their toxicity and potential life-threatening effects. In the AML M3 subtype acute promyelocytic leukemia (APL), ATRA and arsenic trioxide can be used (42). Common side effects of cytarabine include headache, nausea, vomiting, and low blood counts, while less common side effects include flu-like symptoms, loss of appetite, and pain in the hands, feet, and eyes (43). The side effects of daunorubicin's include nausea, vomiting, diarrhea, and hair loss (44). Because of the complexity of AML and its multiple subtypes, the treatment of AML has changed very little over the last few decades. Because of these factors, exploring WGA as a potential therapeutic is worthwhile. The side effects of WGA, such as adverse toxicity and hemagglutination, could be curtailed if administered at low doses.

We show that dietary lectins may be a unique therapeutic tool against hematological malignancies because of their cytotoxic potential and limited toxicity to normal cells and tissues. We characterized the effects on one subtype, OCI-AML3, leaving open the exploration of many other cancer types and conditions, such as drug-resistance and relapse. Characterization of WGA-killing may also lead to more information on novel cell killing pathways. Insights into WGA as a drug-delivery system (45, 46), might also be utilized in combination with our findings to develop potential targeted treatments for hematological malignancies.

## Ethics Statement

Case Western Reserve University's Institution's Animal Care and Use Committee (IACUC) reviewed and approved the Animal Experimentation performed in this study and followed IACUC guide lines and protocol. Mice were maintained in ARC facility at Case Western Reserve University under a 12-h day-night cycle and had *ad libitum* access to food and water. Human samples from normal donors and leukemia patients were obtained from Hematopoietic Stem Cell Core Facility located in Case Western Reserve University. Discarded human blood samples were used in this study and informed consent documents were approved by the University Hospitals Case Medical Center Institutional Review Board.

## Author Contributions

BR: performed experiments, data analysis, and writing. KZ: performed *in vivo* xenograft experiment. AA: performed *in vivo* toxicity experiments. DW: performed some of the *in vitro* experiments. YV: performed some of the *in vitro* experiments. RP: conceived the idea, analyzed data, wrote and edited manuscript.

### Conflict of Interest Statement

The authors declare that the research was conducted in the absence of any commercial or financial relationships that could be construed as a potential conflict of interest.

## Abbreviations

WGA: Wheat germ agglutinin
AML: acute myeloid leukemia
ALL: acute lymphoid leukemia
NHL: non-Hodgkin Lymphoma
sWGA: Succinyl-WGA
LEL: *L. esculentum* lectin
SNA: *S. nigra; N*-acetyl-D-neuraminic (sialic) acid
GlcNAc: *N*-acetyl-D-glucosamine.
